# Composition-Dependent Wettability, Moisture Sorption and Adhesion of HPMC, Carbopol and Kollidon Binary Matrices to a Model Mucin Layer: An In Vitro Study

**DOI:** 10.3390/polym18212681

**Published:** 2026-10-29

**Authors:** Monika Rojewska, Klaudia Szelejewska, Karolina Fikus, Emilia Jakubowska, Tomasz Osmałek, Krystyna Prochaska

**Affiliations:** 1 Institute of Chemical Technology and Engineering, Faculty of Chemical Technology, Poznan University of Technology, ul. Berdychowo 4, 61-131 Poznan, Poland; 2 Department of Pharmaceutical Technology, Faculty of Pharmacy, Poznan University of Medical Sciences, 3 Rokietnicka, 60-806 Poznan, Poland

**Keywords:** functional polymer matrices, mucin–polymer interactions, wettability, surface free energy, hydration, mucin-layer adhesion

## Abstract

This study systematically evaluates the composition-dependent wettability, apparent surface free energy, moisture sorption, and adhesion to a model mucin layer of binary polymer matrices composed of hydroxypropyl methylcellulose (HPMC), Carbopol 974P NF, and Kollidon VA 64, complemented by liquid uptake measurements of the individual polymer powders. API-free compressed discs containing three binary polymer combinations at weight ratios of 1:2, 1:1, and 2:1 were investigated using contact angle measurements, surface free energy estimation, liquid uptake analysis, moisture sorption studies, scanning electron microscopy, and in vitro detachment-force measurements. The results revealed distinct composition-dependent responses. HPMC exhibited limited initial wettability, with a water contact angle of 103.4°, and the lowest moisture uptake (14.8% after 96 h at 25 °C and 85% RH). Incorporation of Carbopol improved wettability and increased moisture sorption, with the HPMC:Carbopol 1:2 matrix exhibiting a water contact angle of 44.4° and a moisture mass gain of 32.7%. HPMC:Carbopol blends exhibited reproducible detachment forces against the model mucin layer, ranging from approximately 1.2 to 1.5 N. In contrast, Kollidon-containing matrices exhibited favorable initial wetting, but selected Kollidon-rich formulations showed insufficient cohesion during contact with the mucin layer, preventing reliable detachment-force measurements. These findings indicate that polymer composition influences the balance between early wettability, moisture uptake, and measurable adhesion under the investigated in vitro conditions. Among the tested formulations, HPMC:Carbopol 1:2 showed a favorable combination of these properties, supporting its further evaluation as a potential mucoadhesive matrix.

## 1. Introduction

Functional polymer matrices capable of controlled hydration, interfacial interaction and adhesion remain important materials for the development of advanced pharmaceutical platforms and controlled release systems [1,2,3,4,5]. In this context, various polymeric matrix systems have emerged as promising and versatile platforms for the design and formulation of advanced dosage forms [6,7]. Among these, mucin-interacting and mucoadhesive polymeric materials have garnered particular attention due to their remarkable potential to act as effective carriers for sustained and site-specific drug delivery [8,9,10]. Mucoadhesive polymers adhere to the mucosal surface through a combination of physical and chemical interactions, primarily involving hydrogen bonding, van der Waals forces, and electrostatic interactions with mucin glycoproteins present in the mucus layer. These interactions prolong the residence time of the formulation at the site of application and can enhance drug absorption across the mucosal membrane. Therefore, the investigation of surface and hydration-related properties of polymer blends in mucin-containing environments constitutes a crucial step in understanding their biointerfacial functionality, as it enables detailed characterization of the interactions between these materials and mucosal surfaces [11,12]. Surface and microstructural characterization of polymer blends is essential for understanding their composition-dependent interfacial behavior [13,14,15]. In parallel, contact angle measurements are employed to evaluate apparent wettability and interfacial liquid spreading, offering valuable information about the interfacial behavior of the polymer matrices [16,17]. Understanding key surface parameters such as surface free energy (SFE) and wettability is crucial for the rational selection and modification of polymer blend compositions. These characteristics are closely related to hydration, mucin-layer adhesion and matrix performance, ultimately determining the efficiency of controlled drug release at the site of administration [18,19]. SFE is commonly calculated using models such as the Owens–Wendt method, which separates the total surface free energy into polar and dispersive components based on contact angle measurements with different probe liquids [20,21]. This method has been extensively validated in pharmaceutical research for characterizing interfacial properties that govern both mucoadhesion and drug release kinetics [22]. The integration of surface imaging techniques with contact angle and surface free energy analysis provides essential quantitative data that guide the design and optimization of polymer blends in terms of composition, microstructure, and processing parameters. Such an approach supports the development of functional polymer matrices with tunable hydration, adhesion and biointerfacial behavior [23].

Hydroxypropyl methylcellulose (HPMC) is a widely used polymer in mucoadhesive drug delivery systems due to its favorable physicochemical properties, biocompatibility, and regulatory acceptance. Its hydroxyl-rich structure enables hydrogen bonding with mucin glycoproteins, enhancing adhesion and prolonging mucosal residence time. Upon hydration, HPMC swells to form a viscous gel that supports controlled and sustained drug release [24]. Moreover, the ease of tailoring its molecular weight and viscosity grades allows for fine-tuning of the mucoadhesive and release characteristics, which makes it a highly adaptable excipient for mucoadhesive dosage forms [25,26]. In the present study, HPMC was combined with Carbopol 974P NF and Kollidon VA 64, representing three distinct functional polymer chemistries: a non-ionic gel-forming cellulose ether, an ionizable crosslinked poly(acrylic acid), and a hydrophilic vinylpyrrolidone/vinyl acetate copolymer, respectively. Carbopol 974P NF provides ionizable carboxyl groups, high swelling capacity and pH-dependent gel-forming behavior, whereas Kollidon VA 64 contributes hydrophilicity and film-forming properties, although its contribution to matrix cohesion may depend strongly on hydration conditions. The systematic investigation of these binary polymer blends represents an important strategy for defining composition–property relationships in functional mucin-interacting matrices.

By combining HPMC with polymers such as carbomers (e.g., Carbopol^®^), sodium alginate, chitosan, or poly(acrylic acid) derivatives, it is possible to exploit complementary functional properties, including enhanced swelling capacity, improved mucin-layer adhesion, and tailored gel formation. For example, blends of HPMC and carbomers have been shown to exhibit superior bioadhesive performance due to synergistic hydrogen bonding and improved hydration dynamics [27,28], whereas incorporation of chitosan can impart additional bioadhesive interactions with mucin glycoproteins via ionic mechanisms. Such multi-polymer matrices enable modulation of key interfacial parameters, including hydration rate, gel-layer formation, surface polarity, cohesion and adhesion to mucin-rich environments, which are often unattainable with single-component systems. Consequently, the use of HPMC-based blends facilitates the development of dosage forms with enhanced mucoadhesive performance, prolonged therapeutic action, and improved patient compliance [29]. Although the wettability, swelling, sorption, and surface morphology of selected mucoadhesive polymers and their blends have been investigated in previous studies [14,17,19,22,30], the influence of polymer weight ratio on the physicochemical behavior of selected binary matrices remains insufficiently characterized.

In our previous study [30], HPMC, Carbopol 974P NF, Kollidon VA 64, and selected HPMC-based binary blends at a fixed 1:1 weight ratio were investigated in terms of wettability, surface free energy, mucoadhesion, moisture sorption, and hydration-related dimensional changes using surface dissolution imaging (SDi2). While these findings provided a basis for understanding the behavior of individual polymers and equimass blends, the effects of varying polymer proportions and the properties of the Kollidon binary system were not examined.

Therefore, the present study extends our previous research by systematically evaluating three binary polymer combinations, namely HPMC:Carbopol, HPMC:Kollidon, and Kollidon:Carbopol, at 1:2, 1:1, and 2:1 weight ratios. The main objective was to determine how changes in polymer composition affect apparent wettability, surface free energy, moisture sorption, and adhesion to a model mucin layer under defined in vitro conditions. Additional liquid uptake measurements of individual polymer powders and SEM characterization of compressed matrices were performed to provide complementary information on their physicochemical and morphological properties. Selected previously published data [30] were included as reference datasets to enable comparison with the newly investigated formulations. The novelty of this work lies in the extended composition-dependent analysis, including the previously unexamined Kollidon system, and the identification of formulation-dependent differences in wetting, moisture uptake, and mucin-layer adhesion relevant to the selection of polymer matrices for further mucoadhesive applications.

## 2. Methods

### 2.1. Materials

HPMC and mucin from porcine stomach type II (product No. M2378) were provided by Sigma Aldrich (Merck, Poznan, Poland). According to the manufacturer’s Certificate of Analysis (batch No. 0000575391), the mucin was supplied as a light-brown powder with a bound sialic acid content of 0.6% (expressed as N-acetylneuraminic acid). The material was stored at 2–8 °C according to the manufacturer’s recommendations. Hydroxypropyl methylcellulose (HPMC; Sigma-Aldrich, catalogue no. H7509, batch no. BCCP4200), with an approximate molecular weight of 86 kDa and a specified viscosity range of 2600–5600 mPa·s (2% aqueous solution at 20 °C), was used. According to the manufacturer’s Certificate of Analysis, the batch-specific Brookfield viscosity was 2919 mPa·s, determined in a 2% aqueous solution at 20 °C. Kollidon VA 64 fine (hereinafter referred to as ‘Kollidon’) was a generous gift from BASF (Ludwigshafen, Germany). Carbopol 974P NF was obtained from Lubrizol (Poland) company (hereinafter referred to as ‘Carbopol’). The chemical structures of the examined polymers are included in Table S1 (Supplementary Material), while their pharmaceutical applications and physicochemical characteristics have been discussed in our previous studies [17,22]. The simulated saliva fluid was used in the study as the medium [31]. To evaluate the influence of mucin on the physicochemical behavior of the polymer matrices, simulated saliva fluid (SSF) and its modified form containing 0.1% mucin (SSF_muc_) were utilized. SSF and SSFmuc were prepared using the same underlying electrolyte composition, with the latter additionally containing 0.1% (*w*/*v*) mucin. This experimental approach enabled a comparative assessment of wetting behavior in simulated salivary environments without and with mucin addition. The pH values of all testing liquids were measured before the experiments, and their compositions and pH values are provided in Supplementary Table S2. Mucin stock solutions (2% *w*/*v*) were prepared either on the day of the experiment or up to one day in advance and stored at 5 °C to ensure stability. The pH values of all media were measured at 22 ± 1 °C before the experiments. The 2% mucin medium prepared in distilled water was not pH-adjusted and was used as a model hydrated mucin dispersion.

### 2.2. Preparation of Polymeric Discs

Mucoadhesive polymer discs (13 mm diameter) were prepared by direct compression of 200 ± 5 mg powder blends at 2 MPa using a hydraulic press (2 Ton High Profile E-Z Press™, International Crystal Laboratories, Garfield, NJ 07026, USA) for 60 s. The nominal compression pressure of 2 MPa was calculated based on the target compression force and the cross-sectional area of the discs. This pressure was selected based on previous experimental experience, which indicated that discs prepared under these conditions retained sufficient integrity for handling without visible disintegration. During manual compression, the corresponding pressure-gauge indication was monitored and maintained at the same level for all formulations. Binary polymer mixtures were prepared at weight ratios from 1:2 to 2:1. Table S3 (Supplementary Material) summarizes the compositions of the evaluated formulations. The polymer powders were accurately weighed according to the required weight ratios and sieved using sieves with nominal aperture sizes of 0.71 and 1.0 mm. The prepared powder mixtures were subsequently blended using a mechanical shaker for 10 min prior to direct compression. All samples were stored in a desiccator at ambient conditions until further testing.

Selected experimental data for individual polymers and equimass HPMC:Carbopol and HPMC:Kollidon blends (1:1, *w*/*w*), previously published in our study [30], were reused as reference datasets for comparison with the newly investigated formulations. The new experimental series included HPMC-based blends at 1:2 and 2:1 weight ratios, as well as Kollidon:Carbopol blends at 1:2, 1:1, and 2:1 ratios. The reference discs were prepared using the same nominal compression conditions (200 mg, 13 mm diameter, 2 MPa), providing a common methodological basis for comparison. The origin of previously published data is identified in the corresponding tables and figure captions. A detailed comparison of previously published reference datasets [30] and newly generated experimental data is provided in Supplementary Table S6.

**Figure 1:**
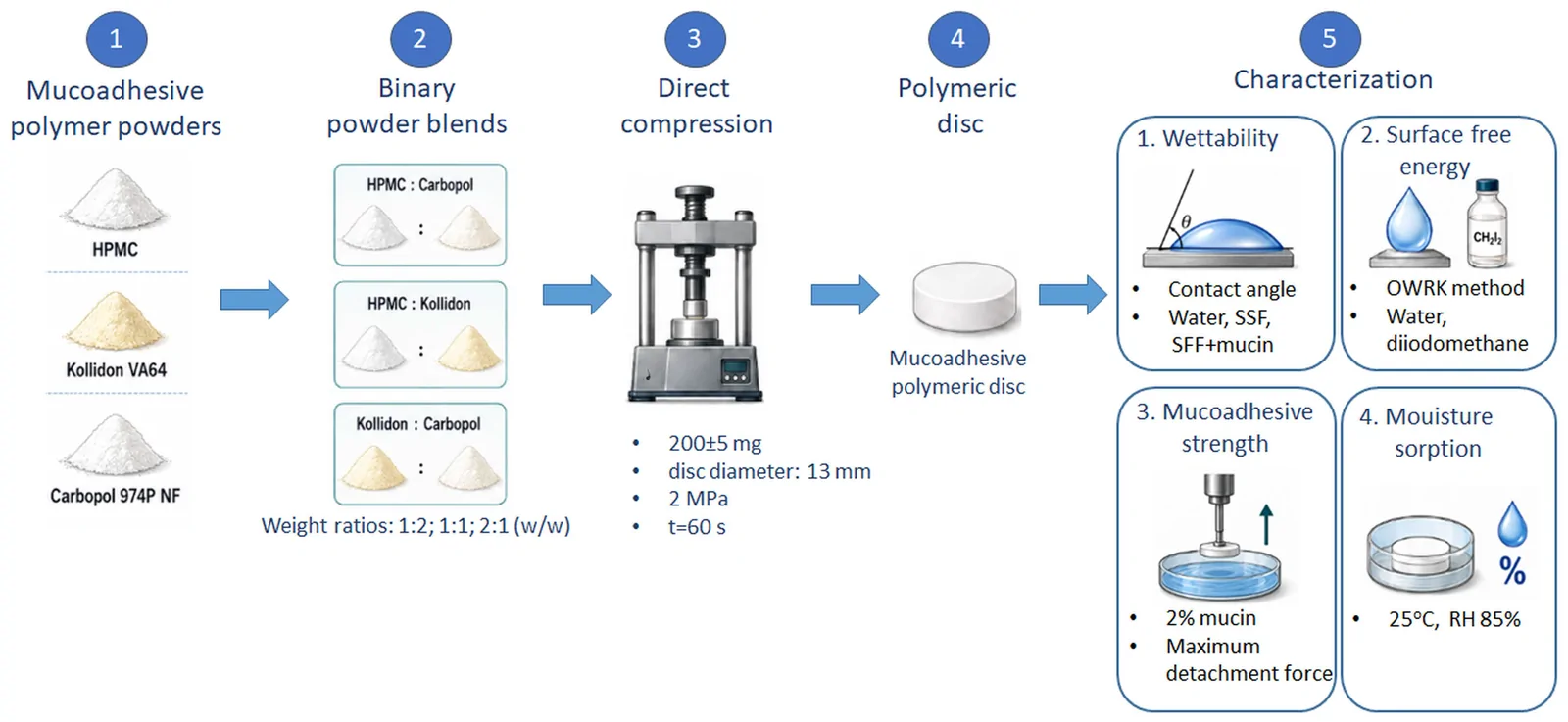
Schematic representation of the experimental workflow used for the preparation and characterization of mucoadhesive polymer discs.

The overall workflow of disc preparation and subsequent physicochemical and mucoadhesive characterizations is schematically presented in Figure 1.

### 2.3. Liquid Uptake Measurements of Individual Polymer Powders

Sorption measurements of individual polymer powders were carried out using the liquid penetration method with a Sigma 700/701 tensiometer (KSV Instruments Ltd., Helsinki, Finland). HPMC, Carbopol 974P NF, and Kollidon VA 64 powders were tested in distilled water, simulated saliva fluid containing mucin (SSF_muc_), and 2% (*w*/*v*) mucin solution. The detailed composition of the investigated media is described in Section 2.1. A powder sample of 200 ± 5 mg was placed in a measuring vessel. To standardize powder-bed preparation, the vessel was manually tapped 50 times with its base against the laboratory benchtop, allowing the powder to settle before measurement. The same tapping procedure was applied to all samples and replicates. Subsequently, the vessel was suspended on the hook of the calibrated microbalance. The vessel containing the powder was automatically lowered until contact with the liquid surface was achieved. The mass of liquid penetrating into the powder bed was continuously recorded for 300 s. The measurements were performed to evaluate the initial liquid uptake capacity and hydration behavior of the individual polymers prior to disc preparation. Each measurement was repeated six times for each polymer–medium system (*n* = 6).

Although the tapping procedure was standardized, differences in particle size, morphology, and packing behavior may have resulted in variations in powder-bed porosity between polymers. Since bed porosity and bulk density were not independently determined, the recorded uptake profiles were interpreted as apparent liquid penetration behavior rather than intrinsic polymer hydration kinetics.

### 2.4. Measurement of Wettability

Apparent contact angles of the polymeric discs were measured using the sessile drop technique with an optical tensiometer (Attension Theta Lite, Biolin Scientific, Espoo,, Finland). Liquid droplets were automatically dispensed onto the disc surface using a precision capillary system. The droplet shape was recorded with a video capture system and analyzed by fitting the experimental contour to the theoretical Young–Laplace model. For each formulation, six replicates were performed to ensure reproducibility and statistical reliability. For the presentation of dynamic water wettability, a representative contact angle curve was selected from six replicate measurements for each formulation, based on its similarity to the typical time-dependent behavior observed in the experimental series.

Statistical analysis of contact angle values was performed for the 15 s time point, which was selected as representative of the early dynamic wetting stage. For each wetting medium, one-way ANOVA followed by Tukey’s post hoc test was used to compare the investigated formulations. Additionally, two-way ANOVA was performed separately for each binary polymer family to evaluate the effects of weight ratio, wetting medium, and their interaction. Differences were considered statistically significant at *p* < 0.05. All statistical analyses were performed using Python (version 3.13.5) with the statsmodels package.

### 2.5. OWRK Method

The Owens–Wendt–Rabel–Kaelble (OWRK) method was applied to estimate the apparent surface free energy (SFE) of the investigated polymeric matrices using contact angles measured with two probe liquids, water and diiodomethane (Table 1). Contact angles measured 1 s after droplet deposition were used for the OWRK calculations. The total SFE was calculated as the sum of its polar and dispersive components. This early measurement time was selected to minimize the effects of progressive hydration, liquid penetration, and swelling on the apparent contact angles. However, the OWRK approach assumes an idealized solid surface, whereas the compressed polymeric matrices investigated in this study exhibit surface heterogeneity and may undergo hydration, swelling, and liquid penetration during measurement. These processes can affect the apparent contact angle and introduce uncertainty into the calculated SFE values. Therefore, the obtained results were considered comparative, model-dependent estimates of early-stage surface energetics rather than equilibrium thermodynamic surface properties.

The method calculates the surface free energy components of the solid using a mathematical model based on Young’s equation and the work of adhesion.

(1) $${\left(\gamma_{\mathrm{sv}}^{d}\right)}^{0.5}=\frac{\gamma_{d}\left(\cos\theta_{d}+1\right)-\sqrt{\frac{\gamma_{d}^{p}}{\gamma_{w}^{p}}}\cdot \gamma_{w}(\cos\theta_{w}+1)}{2\left(\sqrt{\gamma_{d}^{d}}-\sqrt{\gamma_{d}^{p}\frac{\gamma_{w}^{d}}{\gamma_{w}^{p}}}\right)}$$

(2) $${\left(\gamma_{\mathrm{sv}}^{p}\right)}^{0.5}=\frac{\gamma_{w}\left(\cos\theta_{w}+1\right)-2\sqrt{\gamma_{\mathrm{sv}}^{d}\gamma_{w}^{d}}}{2\sqrt{\gamma_{w}^{p}}}$$

where γ_SV_ is the solid-vapor surface tension, γ_w_ is the water surface tension, γ_d_ is the diiodomethane surface tension and θ is the contact angle, which is established by a tangent to the liquid droplet and the solid surface in the area of intersection of the solid–liquid vapor phases (θ_d_ is the contact angle for diiodomethane, and θ_w_ is the contact angle for liquid (water)). The total surface free energy (SFE) of a solid equals:

(3) $$\mathrm{SFE}=\gamma_{\mathrm{sv}}^{d}+\gamma_{\mathrm{sv}}^{p}$$

where $\gamma_{\mathrm{sv}}^{d}$ is the dispersive part of solid surface tension; $\gamma_{\mathrm{sv}}^{p}$ is the polar part of solid surface tension.

### 2.6. In Vitro Mucoadhesive Strength Measurements Using a Model Mucin Layer

Mucoadhesive strength was determined as the maximum detachment force using a Texture Analyzer (Shimadzu AGS-1kNX, Shimadzu Corporation, Kyoto, Japan). Polymeric discs (200 mg), prepared from individual polymers or binary blends, were placed on a stainless steel probe. A layer covering the bottom of a Petri dish, formed by 2 mL of 2% (*w*/*v*) mucin solution, was used as the adhesive interface. The probe was lowered until the disc contacted the mucin layer and held under a compressive force of 0.1 N for 30 s to allow bond formation. The probe was then withdrawn vertically at a constant speed of 10 mm/min until detachment occurred. The maximum peak force observed during detachment was recorded as the mucoadhesive strength. Data were analyzed using the instrument software from the force–distance curves. Measurements were performed in six replicates for each formulation to ensure reproducibility. In this study, the term “mucoadhesive strength” is used as an operational descriptor of the maximum detachment force measured under the defined in vitro conditions against a hydrated 2% mucin layer. This simplified mucin-layer model does not reproduce the full structural and biological complexity of native mucosal tissue, but it enables comparison of the detachment-force responses of the investigated polymer matrices under standardized conditions. Preliminary control measurements were also performed using water without mucin to assess the magnitude of detachment forces in the absence of the model mucin layer.

### 2.7. Moisture Sorption Measurements at 85% Relative Humidity

Moisture sorption studies were performed using a climate chamber (JeioTech TH-ICH-800, JeioTech Co., Ltd., Daejeon, Republic of Korea ) maintained at 25 °C and 85% relative humidity. Each polymeric disc was weighed to determine its initial mass before the experiment. Samples were placed in open weighing containers inside the climate chamber to allow exposure to controlled humidity. At 24-h intervals for 4 days, discs were removed and reweighed using an analytical balance (RADWAG, Radom, Poland) to assess moisture uptake. For each formulation, five replicate measurements were performed (*n* = 5).

The percentage moisture mass gain (MG) of the polymeric discs after exposure to controlled humidity was calculated using the following equation:

(4) $$\mathrm{MG}\left(\%\right)=\frac{W_{t}-W_{0}}{W_{0}}\cdot 100\%$$

where W_0_ is the initial mass of the disc before exposure to controlled humidity, and W_t_ is its mass after a specified exposure time (t). The final percentage moisture mass gain was determined after 96 h.

### 2.8. SEM

The morphology of individual polymer powders and selected compressed polymeric discs was analyzed by scanning electron microscopy (SEM; VEGA 5135, TESCAN, Brno, Czech Republic). For compressed matrices, representative discs containing HPMC, HPMC:Carbopol, HPMC:Kollidon and Kollidon:Carbopol were analyzed to assess composition-dependent surface microstructure after direct compression. The samples were mounted on conductive adhesive tape and sputter-coated with a thin carbon layer prior to visualization. SEM images were recorded at an accelerating voltage of 13.8 kV and used to compare the morphology of the individual polymer powders.

## 3. Results and Discussion

### 3.1. Morphology and Hydration Baseline of Individual Polymer Powders

**Figure 2:**
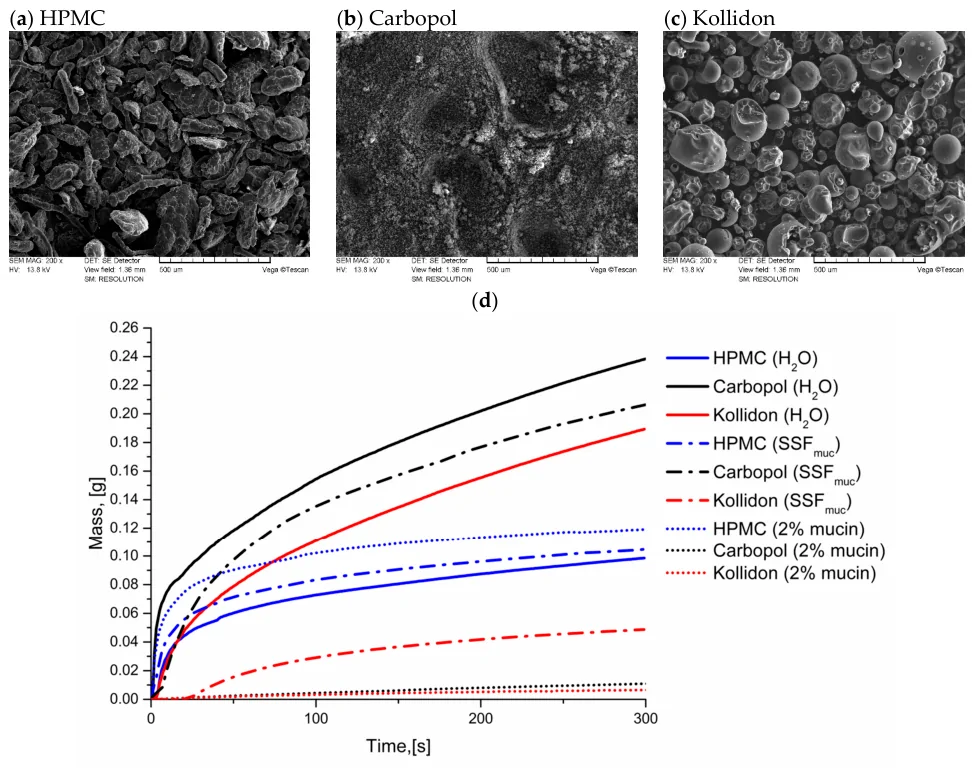
Morphology and liquid uptake behavior of individual polymer powders. SEM images of individual polymer powders (x200): (**a**) HPMC, (**b**) Carbopol, and (**c**) Kollidon; and (**d**) liquid uptake profiles of HPMC, Carbopol 974P NF, and Kollidon powders in water, SSF_muc_, and 2% mucin solution. The curves represent apparent liquid uptake by loose single-polymer powder beds and provide a comparative characterization of the starting materials, without directly representing the hydration behavior of compressed binary matrices (*n* = 6).

Before analyzing the composition-dependent behavior of binary matrices, the individual polymer powders were characterized to establish a physicochemical baseline for further interpretation. This approach is justified because the first stage of mucoadhesion is governed by wetting and hydration of the polymer surface, followed by swelling, chain relaxation, and possible interpenetration with mucin chains [17,19]. Previous studies have also shown that the morphology, surface properties, and sorption capacity of mucoadhesive polymers strongly influence their behavior after compression and their performance in biological media [14,17,22]. SEM images revealed clear differences in the morphology of HPMC, Carbopol, and Kollidon powders (Figure 2a–c). These differences may affect powder packing, capillary penetration of liquids, and the effective surface area available during the early hydration stage. These morphological differences may influence powder-bed packing and capillary liquid penetration. However, the liquid uptake behavior of loose powders cannot be directly extrapolated to compressed binary matrices, in which compaction and polymer blending modify the pore structure, surface organization, and hydration conditions.

The liquid uptake profiles of the individual polymer powders showed pronounced differences depending on polymer type and testing medium (Figure 2d). Under the applied experimental conditions, Carbopol and Kollidon exhibited higher apparent liquid uptake in water than HPMC, whereas the presence of mucin-containing media modified the uptake profiles in a polymer-dependent manner. These differences may reflect variations in polymer wettability, particle hydration, swelling behavior, and capillary penetration into the powder bed. However, the influence of powder packing, interparticle void structure, and liquid physicochemical properties cannot be excluded. Therefore, the recorded curves should be regarded as apparent liquid uptake profiles rather than direct measurements of intrinsic polymer hydration mechanisms.

Although a standardized tapping procedure was applied during powder-bed preparation, differences in particle size, packing, porosity, and capillary structure may also have influenced liquid penetration kinetics and cannot be excluded as contributing factors. Importantly, these experiments were performed on individual polymer powders and were intended to provide supplementary information on the behavior of the starting materials. They were not used to quantitatively predict the hydration kinetics or structural behavior of compressed binary matrices. The properties of the compressed formulations were evaluated separately through contact angle measurements, dry-surface morphology analysis, moisture sorption experiments, and mucoadhesive strength testing. Together, these complementary measurements provide a broader assessment of composition-dependent matrix performance, without assuming a direct mechanistic equivalence between loose powders and compressed polymer blends.

### 3.2. Microstructure of Compressed Polymeric Discs

**Figure 3:**
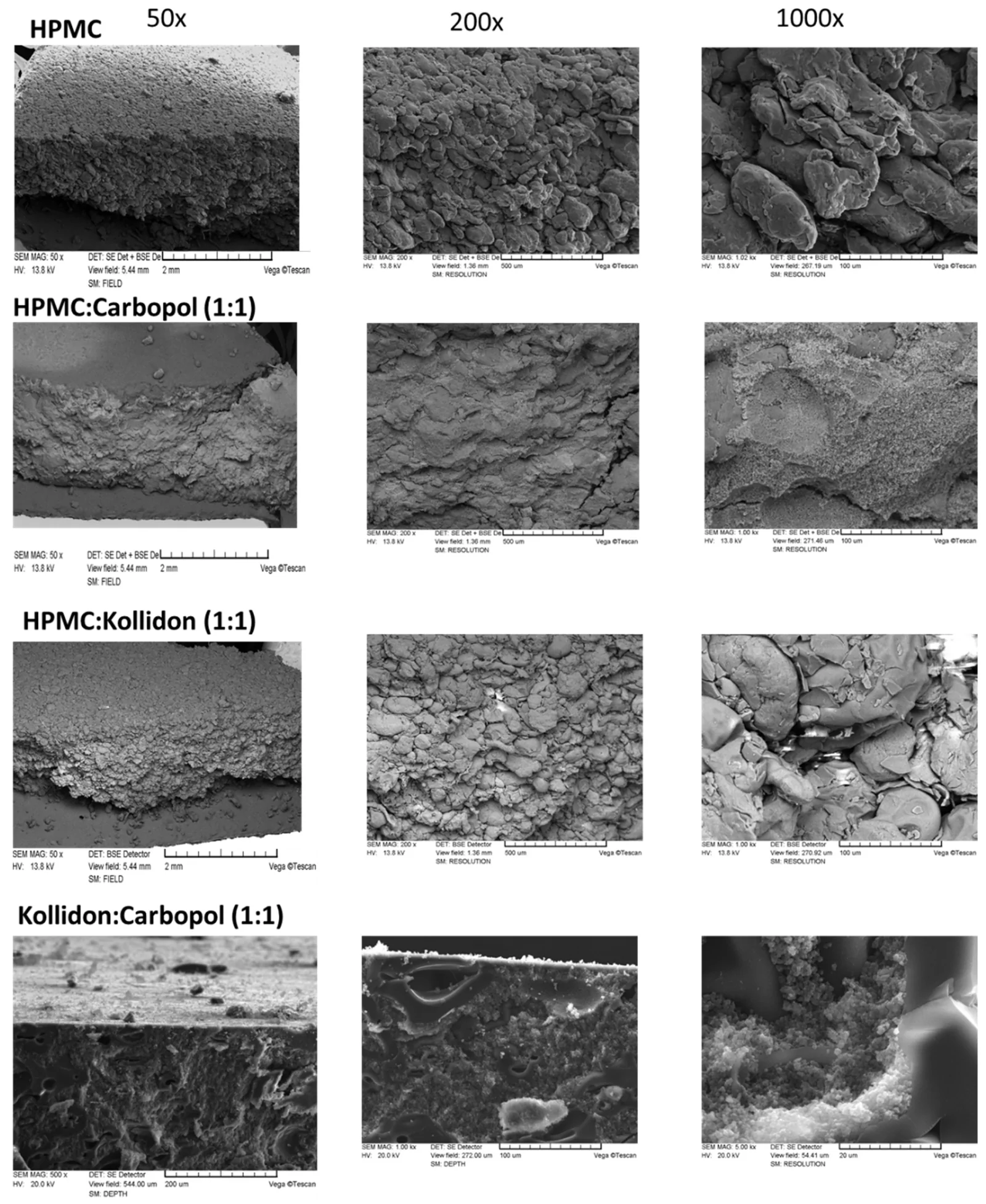
SEM images of compressed polymeric discs prepared at an equal weight ratio. Representative SEM micrographs of HPMC and equimass binary polymeric matrices: HPMC:Carbopol (1:1, *w*/*w*), HPMC:Kollidon (1:1, *w*/*w*), and Kollidon:Carbopol (1:1, *w*/*w*), prepared by direct compression at 2 MPa. The images illustrate qualitative differences in the dry-state morphology of the compressed matrices, including particle-domain organization, surface irregularities, and microstructural heterogeneity.

After evaluating the morphology and liquid uptake behavior of the starting powders, representative compressed discs were analyzed by SEM to determine whether polymer composition affects the microstructure of the matrices formed during direct compression. For this purpose, HPMC and binary matrices prepared at an equal weight ratio, namely HPMC:Carbopol, HPMC:Kollidon, and Kollidon:Carbopol (1:1, *w*/*w*), were selected as representative systems (Figure 3). The complete SEM panel, including all tested weight ratios, is provided in the Supplementary Material (Figure S1).

Although all discs were prepared under the same compression conditions, their surface morphology differed markedly depending on polymer composition. The HPMC matrix showed a granular and layered structure, whereas the HPMC:Carbopol disc appeared more compact and relatively uniform in the examined regions. In contrast, HPMC:Kollidon and Kollidon:Carbopol matrices displayed more heterogeneous domains, visible discontinuities, and less uniform particle organization. These observations indicate composition-dependent differences in the morphology of the compressed matrices. Such differences could potentially influence liquid spreading and penetration during subsequent contact with aqueous media. However, the SEM images were obtained from dry samples and therefore cannot establish the formation, continuity, or mechanical stability of hydrated gel layers.

These morphological differences provide complementary information for interpreting the wettability and mucoadhesive results discussed in the following sections. Surface heterogeneity may contribute to the apparent wetting behavior of compressed polymeric matrices, alongside polymer composition and surface chemistry. Although the discs were prepared under identical nominal compression conditions, differences in their thickness, density, porosity, and mechanical strength may also have influenced the measured properties; however, these parameters were not independently quantified. The supplementary SEM images (Figure S1) further illustrate qualitative changes in dry-state morphology associated with variations in polymer weight ratio. Nevertheless, the present SEM observations do not allow the individual contributions of surface morphology, hydration processes, and polymer–mucin interactions to be distinguished. Any proposed relationships between dry-state microstructure and hydrated-state behavior should therefore be regarded as hypotheses requiring verification through direct measurements under hydrated conditions [17,22].

### 3.3. Wetting Properties of Polymer Blends

#### 3.3.1. Effect of Polymer Composition on Wettability

In the first stage of the study, the wettability of the mucoadhesive matrices containing varying proportions of HPMC, Kollidon, and Carbopol was evaluated.

**Figure 4:**
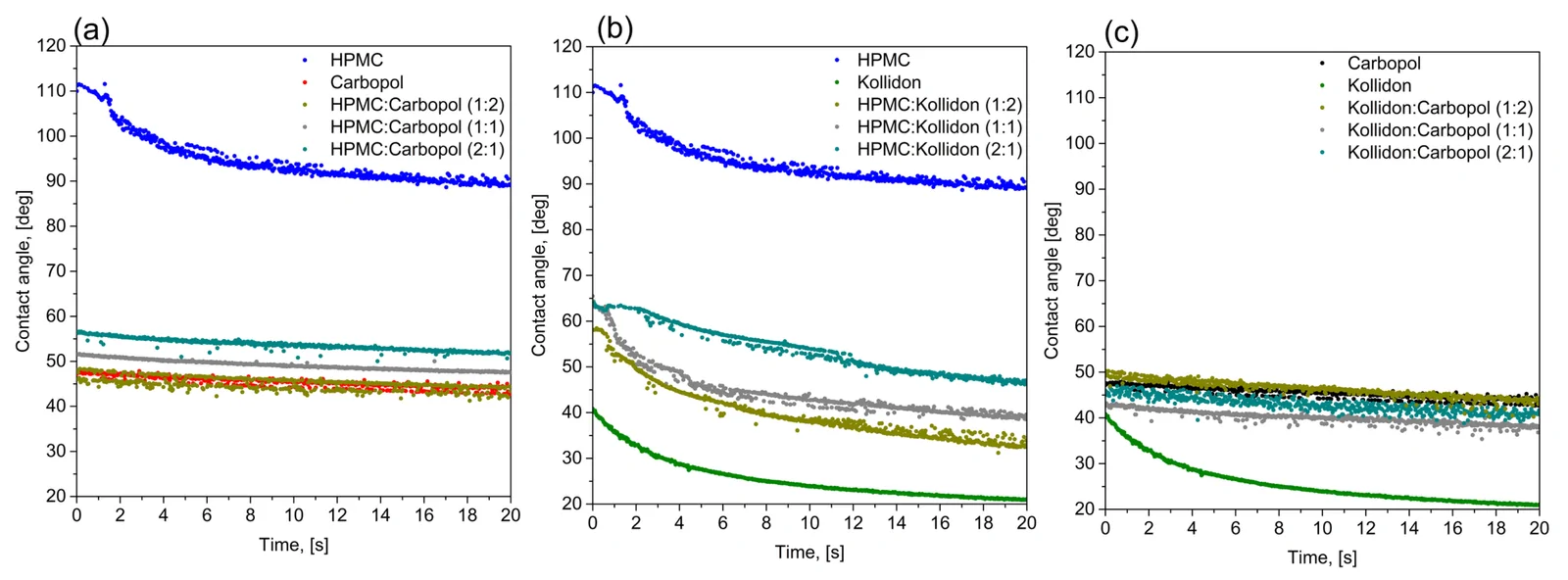
Representative time-dependent water contact angle curves for polymeric matrices prepared at different weight ratios of mucoadhesive polymers (1:2; 1:1; 2:1 *w*/*w*): (**a**) HPMC:Carbopol, (**b**) HPMC:Kollidon, (**c**) Kollidon:Carbopol blends. Data for individual polymers and the HPMC-based 1:1 blends were previously reported in Ref. [30] and are included for comparison with the newly investigated formulations. The curves illustrate representative measurements selected from six experimental replicates performed for each formulation (*n* = 6).

The water wetting curves presented in Figure 4 demonstrate that the incorporation of Carbopol or Kollidon into HPMC-based matrices improved their apparent wettability. HPMC exhibited the highest initial water contact angle among the investigated individual polymers (103.4°), whereas substantially lower values were obtained for the corresponding binary formulations (Table 2).

In the HPMC system, increasing the Kollidon fraction was associated with a progressive decrease in the initial water contact angle, from 62.1° for the HPMC-rich 2:1 blend to 53.9° for the equimass formulation and 51.2° for the Kollidon-rich 1:2 blend. This composition-dependent trend is consistent with the time-dependent water wetting curves shown in Figure 4b. A similar improvement in water wettability with increasing Carbopol content was observed for HPMC matrices (Figure 4a).

Because the investigated matrices are swellable and compositionally heterogeneous, the measured contact angles represent apparent early-stage wetting parameters rather than equilibrium thermodynamic contact angles.

The incorporation of Kollidon into HPMC-based matrices was accompanied by higher apparent SFE values estimated using the OWRK method (Table 2), consistent with the observed improvement in early-stage water wettability. Specifically, for HPMC blends, increasing the Kollidon content from the 2:1 to the 1:2 weight ratio was associated with a decrease in the initial water contact angle from 62.1° to 51.2° (measured 1 s after droplet deposition) and an increase in apparent SFE from 56.7 to 62.6 mJ/m^2^ (Table 2). However, these estimates should be interpreted with caution because the investigated matrices are heterogeneous and susceptible to hydration and swelling during liquid contact. Consequently, the calculated SFE and its polar and dispersive components provide comparative information on surface wetting characteristics under the specified experimental conditions, rather than absolute thermodynamic properties of the polymeric matrices. Moreover, apparent SFE should not be regarded as a direct predictor of mucoadhesive performance, which also depends on hydration-related changes, polymer chain mobility, and the cohesive stability of the hydrated matrix.

The initial water contact angles of Kollidon:Carbopol matrices varied within a relatively narrow range (42.7–48.5°; Table 2), indicating relatively small differences in early-stage water wettability between the investigated compositions. Nevertheless, statistical analysis of the contact angle values measured after 15 s confirmed a significant effect of polymer weight ratio on wettability (Table S5). Furthermore, the significant interaction between polymer ratio and wetting medium indicates that the influence of blend composition on wettability depends on the properties of the contacting liquid.

Among the investigated binary matrices, relatively high apparent SFE values were observed for Kollidon:Carbopol (1:1, *w*/*w*) and HPMC:Carbopol (1:2, *w*/*w*), whereas pure HPMC exhibited a considerably lower apparent SFE. Analyzing the obtained values, it can be concluded that within the HPMC-based binary systems, increasing the HPMC content was generally associated with higher initial water contact angles and lower apparent SFE values, whereas increasing the contribution of Kollidon or Carbopol improved initial water wettability (Figure 4a,b; Table 2). Although Kollidon has a higher surface free energy value than Carbopol, an excess of Kollidon in their binary blends did not result in a consistent increase in the apparent SFE. The values of the dispersive component (γ^d^) were relatively similar across the investigated blends, whereas HPMC alone exhibited a markedly lower dispersive contribution (Table 2). The estimated polar component varied with polymer composition. However, its magnitude and relative contribution to the total apparent SFE should be interpreted cautiously and should not be regarded as direct measures of surface wettability, particularly for heterogeneous and swellable polymeric matrices. In this context, the OWRK-derived surface free energy should be regarded as a comparative descriptor of early-stage surface characteristics rather than as a direct predictor of wettability in mucin-containing media or mucoadhesive performance.

#### 3.3.2. Role of Mucin in Modulating Wettability of Mucoadhesive Polymer Matrices

The presence of mucin, a key glycoprotein component of mucus, may modify the wettability of polymer matrices through changes in both interfacial interactions and the physicochemical properties of the surrounding liquid. Such effects may involve hydrogen bonding, polymer hydration, and pH-dependent ionization, as well as changes in liquid viscosity and surface tension. In the present study, SSF and SSFmuc were prepared using the same electrolyte formulation, providing a comparative basis for assessing changes associated with mucin addition. However, the observed differences in wettability cannot be attributed exclusively to specific polymer–mucin interactions, since mucin may also modify the bulk and interfacial properties of the testing medium.

**Figure 5:**
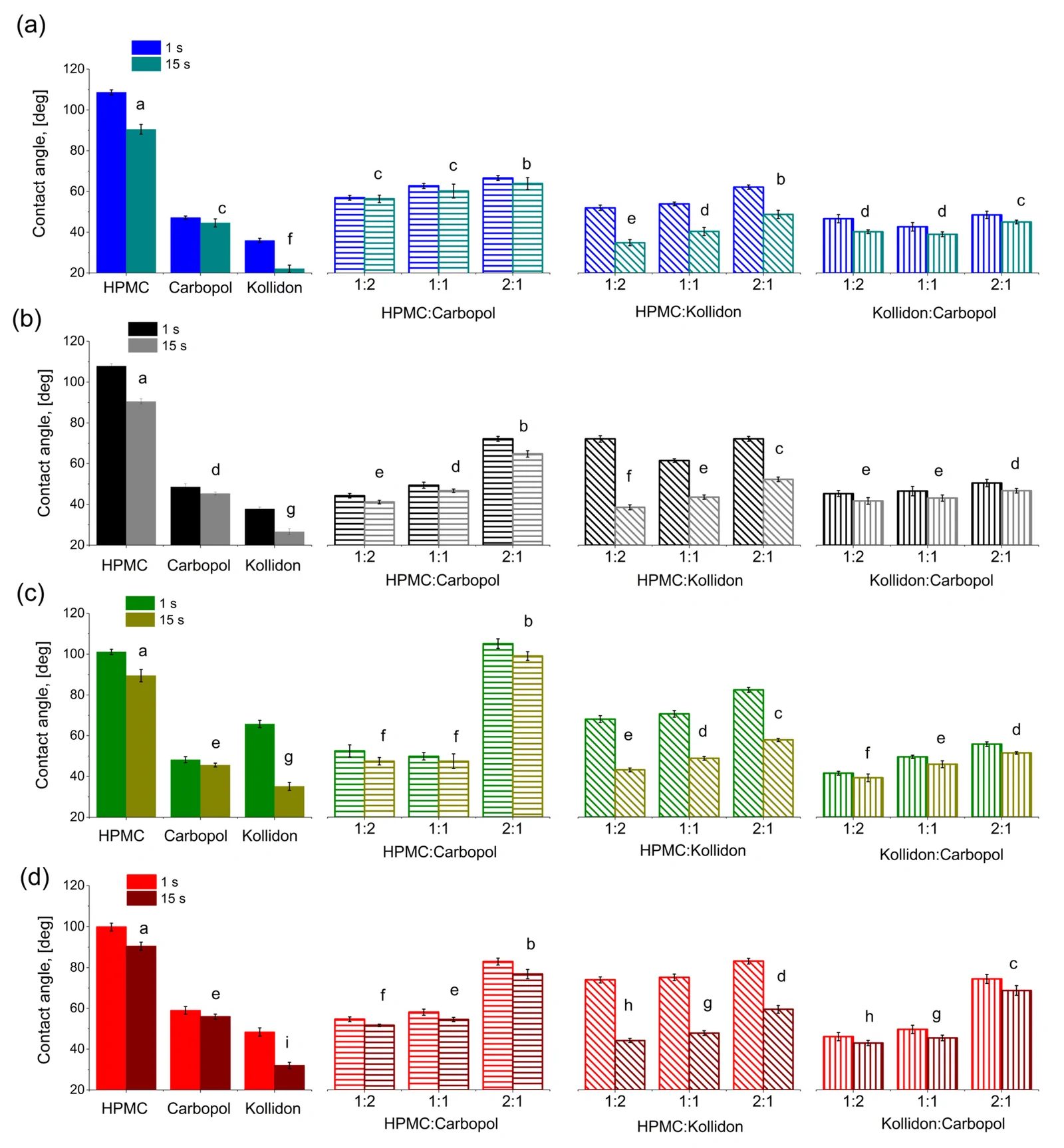
Contact angle values of tested liquids on the surface of individual polymers and binary polymeric matrices. Contact angles were measured 1 s and 15 s after droplet deposition on compressed polymeric discs using (**a**) water, (**b**) simulated saliva fluid without mucin (SSF), (**c**) simulated saliva fluid containing mucin (SSFmuc), and (**d**) 2% mucin solution. The binary matrices HPMC:Carbopol, HPMC:Kollidon, and Kollidon:Carbopol were prepared at weight ratios 1:2, 1:1, and 2:1. Data are presented as means ± SD (*n* = 6). Different lowercase letters indicate statistically significant differences between formulations within the same medium for the 15 s contact angle values; formulations sharing the same letter do not differ significantly according to Tukey’s post hoc test (*p* < 0.05). Previously published data for individual polymers and HPMC-based 1:1 blends [30] were included as reference datasets for comparison with newly investigated compositions.

Figure 5 presents the contact angle values of individual polymers and binary polymer blends measured after 1 s and 15 s of contact with the tested liquids. The data are grouped according to the wetting medium to facilitate direct comparison of the effect of polymer composition within the same liquid environment. The 1 s value represents the first stable measurable contact angle after droplet deposition, whereas the 15 s value reflects the early evolution of liquid spreading and surface hydration before pronounced macroscopic swelling, erosion, or deformation of the discs occurs. This early time window is particularly relevant for mucoadhesive systems, in which rapid wetting and hydration are required to initiate intimate contact with the mucosal model and enable subsequent polymer–mucin interactions.

In all tested media, HPMC exhibited the highest contact angle values, confirming its limited initial wettability. The incorporation of Carbopol or Kollidon generally reduced the contact angle, indicating improved liquid spreading on the surface of binary matrices. This effect was particularly visible for HPMC-based blends, where increasing the contribution of Carbopol or Kollidon enhanced wettability compared with HPMC alone. In contrast, Kollidon:Carbopol matrices showed relatively similar contact angle values across the investigated weight ratios, suggesting that the wettability of these systems was less sensitive to changes in component proportion. The effect of polymer ratio became more pronounced in mucin-containing media. However, changes in the apparent contact angle may reflect a combination of polymer surface properties, hydration behavior, and mucin-induced changes in liquid properties, rather than specific polymer–mucin interactions alone. In particular, HPMC-rich matrices tended to exhibit higher contact angle values than Carbopol- or Kollidon-rich systems, indicating a composition-dependent wetting response under the investigated conditions.

Statistical analysis of the 15 s contact angle values confirmed that formulation composition significantly affected wettability in all tested media (one-way ANOVA, *p* < 0.001, see Table S4). In each panel, formulations marked with the same lowercase letter do not differ significantly, whereas formulations marked with different lowercase letters differ significantly according to Tukey’s post hoc test (*p* < 0.05); in the adopted lettering convention, earlier letters in the alphabet correspond to higher mean contact angle values. Tukey’s post hoc test showed that HPMC formed a separate statistical group in each medium and exhibited the highest contact angle values, confirming its limited wettability compared with the other polymers and binary matrices. In contrast, Kollidon generally belonged to the lowest contact angle groups, indicating more effective liquid spreading on its surface; however, this improved wettability should not be directly interpreted as better formulation performance because Kollidon-rich systems also showed reduced structural stability in mucin-containing media. Among HPMC:Carbopol matrices, the HPMC-rich formulation, HPMC:Carbopol 2:1, consistently showed significantly higher contact angle values than the 1:1 and 1:2 systems, particularly in mucin-containing media. This indicates that increasing the HPMC fraction reduced the early wetting ability of the matrix, whereas a higher Carbopol contribution promoted liquid spreading. A similar composition-dependent trend was observed for HPMC:Kollidon matrices, where contact angle values increased with increasing HPMC content. Thus, the Kollidon-rich HPMC:Kollidon (1:2 *w*/*w*) system showed better wettability (lower contact angle) than the HPMC-rich (2:1 *w*/*w*) formulation, particularly after 15 s of contact with the investigated liquids (Figure 5a–d). This indicates more effective apparent wetting of the Kollidon-rich blend under these conditions. For Kollidon:Carbopol matrices, the effect of the ratio was also statistically significant, with the 2:1 system showing higher contact angle values than the 1:1 and 1:2 systems, especially in 2% mucin solution. These results indicate that the effect of polymer ratio on wettability was strongly dependent on both polymer pair and liquid composition.

Overall, the Tukey grouping confirmed that the observed differences in contact angle were not only numerical but statistically significant, supporting the conclusion that early wetting of the investigated matrices depends on polymer composition, component ratio, and the presence of mucin in the wetting medium (Table S5).

#### 3.3.3. Wettability of HPMC:Carbopol Polymer Blends

For HPMC:Carbopol blends with weight ratios of 1:2 and 1:1, comparable contact angle values were observed, regardless of the wetting liquid applied. These values consistently fell within the range of 40° to 50°. In contrast, higher contact angle values were observed when HPMC was present in excess, particularly in mucin-containing media. In these systems, contact angle values ranged between 60° and 80°.

In a matrix with an excess of HPMC, wetting with mucin-containing saliva resulted in an almost twofold increase in contact angle compared to the 1:2 and 1:1 HPMC:Carbopol blends. Additionally, the application of a 2% mucin solution to the 2:1 blend increased the contact angle by approximately 30% relative to the other compositions. In contrast, when water was used as the wetting agent, the effect of blend composition on contact angle values was less pronounced.

These observations suggest that the wetting behavior of HPMC blends depends on both polymer composition and the properties of the testing medium. The enhanced wettability of Carbopol-rich blends in mucin-containing media may be associated with the hydrophilic character of Carbopol and its ionizable carboxyl groups, which can influence polymer hydration and interfacial behavior. Hydrogen bonding may contribute to polymer–mucin interactions, whereas the possible electrostatic contributions depend on the ionization conditions and cannot be established from the present measurements. The investigated mucin-containing media differed in pH (6.7 for SSFmuc and 4.3 for the 2% mucin solution), which may influence the ionization of Carbopol carboxyl groups and the charge characteristics of mucin. Under these conditions, direct electrostatic attraction between Carbopol and mucin cannot be assumed. However, the contribution of mucin-induced changes in liquid properties cannot be excluded, and the observed contact angle differences should not be considered direct evidence of specific polymer–mucin interactions. These observations may reflect the complementary properties of the two polymers: HPMC has gel-forming characteristics, whereas Carbopol contains ionizable carboxyl groups and exhibits pronounced affinity for water. However, the contribution of hydrogen bonding, polymer chain interpenetration, or electrostatic interactions to the observed wetting behavior was not directly established in this study. Consistent with these observations, Perioli et al. [32] reported differences in swelling and mucoadhesive properties among buccal films prepared using various mucoadhesive polymers, including HPMC and carbomers.

#### 3.3.4. Wettability of HPMC:Kollidon Polymer Blends

The wettability of HPMC blends depended on polymer composition, the wetting medium, and the measurement time. The water wetting curves (Figure 4b) and contact angle values measured 1 s after droplet deposition (Table 2) showed that increasing the Kollidon fraction improved the initial apparent wettability of the matrices. The water contact angle decreased from 62.1° for HPMC:Kollidon (2:1, *w*/*w*) to 53.9° for the 1:1 blend and 51.2° for the Kollidon-rich HPMC:Kollidon (1:2, *w*/*w*) formulation. In mucin-containing media (Figure 5c,d), the Kollidon-rich formulation also generally exhibited lower contact angles than the HPMC-rich blend, particularly after 15 s of contact. However, the magnitude of these differences depended on the wetting medium and measurement time.

The observed composition-dependent wetting behavior may be associated with differences in the chemical structure and surface characteristics of HPMC and Kollidon VA 64. HPMC contains hydroxyl groups capable of participating in hydrogen bonding, whereas Kollidon VA 64, a copolymer of vinylpyrrolidone and vinyl acetate, contains carbonyl groups that may contribute to interactions with aqueous media. Previous studies on HPMC-based films incorporating a vinylpyrrolidone/vinyl acetate copolymer have demonstrated that the addition of this copolymer can modify the moisture permeability and mechanical properties of the resulting films [33]. Surface morphology and heterogeneity may also affect the apparent contact angles. Moreover, hydrogen bonding and other intermolecular interactions can contribute to polymer–mucin interactions [34,35]. Nevertheless, the present contact angle measurements and dry-state SEM observations do not allow these mechanisms to be distinguished or the formation and stability of hydrated interfacial layers to be established. Improved wettability should therefore not be regarded as direct evidence of enhanced matrix cohesion under hydrated conditions.

The apparent surface free energy values estimated using the OWRK method (Table 2) were consistent with the initial water wettability trend. The Kollidon-rich HPMC (1:2, *w*/*w*) formulation exhibited an apparent SFE of 62.6 mJ/m^2^, with a polar component of 13.9 mJ/m^2^, corresponding to approximately 22% of the total value. In comparison, the HPMC-rich (2:1, *w*/*w*) formulation exhibited a lower apparent SFE of 56.7 mJ/m^2^, with a polar contribution of 8.7 mJ/m^2^ (approximately 15.3%). However, these OWRK-derived parameters should be interpreted as comparative, model-dependent estimates of early-stage surface characteristics rather than equilibrium thermodynamic properties or direct evidence of specific interactions with mucin.

Overall, the results indicate that increasing the Kollidon fraction generally promotes apparent wettability under the investigated conditions, although improved wetting should not be equated with greater cohesion or stability of the matrices under hydrated conditions.

#### 3.3.5. Wettability of Kollidon:Carbopol Polymer Blends

A similar trend was also observed in the Kollidon:Carbopol blends, where an excess of Kollidon led to decreased wettability of the matrix by mucin-containing fluids (2:1 *w*/*w*; Figure 5c,d). In contrast, regardless of the wetting medium used, the 1:1 and 1:2 (*w*/*w*) Kollidon:Carbopol blends exhibited very similar contact angle values, indicating comparable surface wettability. This behavior may be associated with the hydrophilic and ionizable nature of Carbopol, whose carboxyl groups can influence hydration and may participate in hydrogen bonding. The ionization of Carbopol and mucin depends on the pH of the surrounding medium (6.7 for SSFmuc and 4.3 for the 2% mucin solution). Previous studies have demonstrated that the structural and viscoelastic properties of Carbopol-based hydrogels depend on pH [36], while spectroscopic investigations have provided evidence of interactions between poly(acrylic acid) and mucin [37]. Under near-neutral conditions, both Carbopol and mucin may carry negative charges; therefore, direct electrostatic attraction between them cannot be assumed. The contribution of specific molecular interactions was not directly established in this study. In addition, hydrogen bonding between the functional groups of Kollidon VA 64 and Carbopol, together with hydration-induced changes in surface organization, may influence the wetting behavior of these blends. Consequently, the observed differences cannot be unequivocally attributed to specific polymer–mucin interactions, as changes in liquid properties and matrix hydration may also contribute to the apparent contact angle values. Indeed, neutral polymers such as Kollidon are known to exhibit weak and nonspecific interactions with mucin compared with ionic or highly mucoadhesive polymers [38], and Kollidon has been shown to adsorb to biomimetic surfaces, modulating surface properties and potentially impeding direct mucin binding [39]. Furthermore, theoretical descriptions of mucoadhesion emphasize that steric and electrostatic barriers at interfaces can reduce effective contact and adhesion with mucin layers. Previous studies on mucoadhesive polymer blends containing Carbopol 974P NF, Kollidon VA 64, and other polymers have demonstrated that formulation composition influences sorption and mucoadhesive properties [40], supporting the importance of polymer composition in determining the functional behavior of such systems. Thus, the observed wetting behavior cannot be explained solely by the hydrophilicity of individual components and may also reflect composition-dependent surface characteristics and the properties of the wetting medium. These findings highlight the necessity of considering the complex physicochemical and morphological factors governing polymer blend surfaces when evaluating their wetting properties, especially in systems designed for controlled drug delivery or bioadhesion where mucin interactions are critical.

Two-way ANOVA performed separately for each binary polymer family confirmed significant effects of wetting medium and polymer ratio on the 15 s contact angle values. Significant medium × ratio interactions were observed for HPMC:Carbopol and Kollidon:Carbopol matrices, whereas this interaction was not significant for HPMC:Kollidon matrices, indicating a more consistent ratio-dependent wetting response across the tested media. Detailed ANOVA results are provided in Table S5 (Supplementary Material). These statistical results support the interpretation that the investigated matrices exhibit composition-dependent wetting behavior and that their responses vary with the testing medium. However, the statistical significance of these effects does not identify the underlying molecular mechanisms or distinguish specific polymer–mucin interactions from changes in the physicochemical properties of the liquid phase. The greater sensitivity of Carbopol-containing blends to changes in the wetting medium may be attributed, at least in part, to the presence of ionizable carboxyl groups in Carbopol. Previous studies have demonstrated that the ionization and physicochemical behavior of poly(acrylic acid)-based polymers are strongly influenced by the pH of the surrounding medium [17,36,37]. Consequently, changes in the Carbopol fraction may modify the response of these matrices to different wetting liquids. In contrast, HPMC and Kollidon VA 64 are predominantly nonionic polymers, which may contribute to a more consistent influence of the polymer ratio on wettability across the investigated media. Importantly, the nonsignificant medium × composition interaction for HPMC:Kollidon matrices does not indicate that the contact angles were unaffected by the wetting medium, but rather that the influence of polymer ratio on wettability was comparatively consistent across the investigated media.These interpretations remain tentative, as the ionization state and specific molecular interactions were not directly characterized in the present study.

### 3.4. Mucoadhesive Strength of Polymeric Matrices in a Model Mucin Layer

In this study, mucoadhesive strength is used as an operational descriptor of the maximum detachment force measured against a model hydrated mucin layer. Therefore, the obtained values should be interpreted as comparative indicators of mucin-layer adhesion under standardized in vitro conditions, reflecting both interfacial interactions and the cohesive integrity of the polymer matrix during contact with the mucin layer. The assessment of this parameter is particularly useful for polymer blends, where the combination of HPMC, Kollidon and Carbopol may influence the measured adhesion response in a composition-dependent manner. In this study, we evaluated the mucoadhesive properties of polymer matrices with varying weight ratios to determine how each component influences the overall adhesion. For the pure Kollidon disc, as well as for the Kollidon-rich Kollidon:Carbopol blend (2:1, *w*/*w*), mucoadhesive strength could not be determined. In both cases, contact with the 2% mucin solution caused rapid swelling, partial dissolution, and loss of matrix cohesion, which prevented the formation of stable adhesive contact and made reliable detachment-force measurement impossible. In contrast, Kollidon:Carbopol matrices containing higher Carbopol fractions retained sufficient structural integrity during the test and allowed determination of the detachment force. The measurable adhesion observed for the 1:2 and 1:1 blends suggests that Carbopol partially compensated for the limited cohesion of Kollidon-rich systems. This behavior may be associated with the crosslinked poly(acrylic acid) structure of Carbopol and the presence of ionizable carboxyl groups, which can influence hydration and interfacial behavior. However, the contribution of specific polymer–mucin interactions was not directly established in the present study. Therefore, the Kollidon:Carbopol system shows a clear composition-dependent transition: excess Kollidon compromises matrix stability, whereas increasing the Carbopol fraction allows reliable determination of the detachment force under the investigated conditions. The inability to determine detachment force for Kollidon-rich matrices should therefore not be treated as a methodological failure, but as a material response indicating insufficient cohesion of the hydrated matrix under mucin-layer contact.

**Figure 6:**
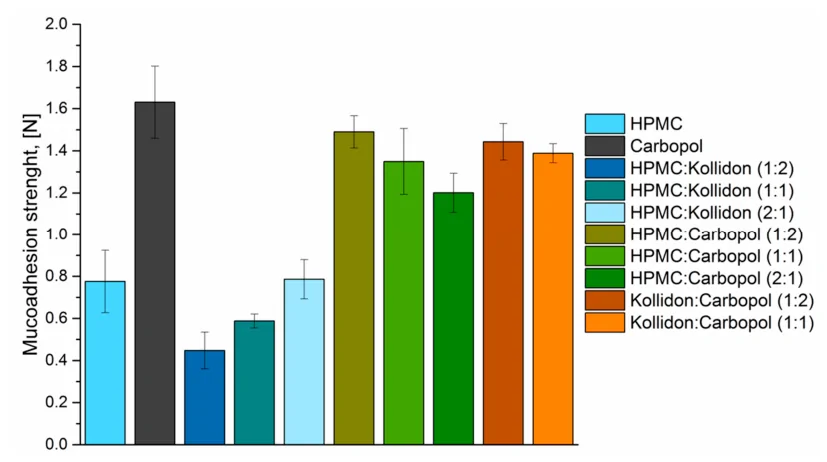
Maximum detachment forces measured for individual polymer matrices and binary blends against a model 2% (*w*/*v*) mucin layer under standardized in vitro conditions. Previously published data for individual polymers and HPMC-based 1:1 blends [30] were included as reference values for comparison with newly investigated formulations. Error bars represent standard deviations. Preliminary mucin-free control experiments conducted using water under identical measurement conditions yielded substantially lower detachment forces than those recorded in the presence of mucin, with some values approaching zero (*n* = 6).

Figure 6 shows the mucoadhesive forces measured using a texture analyzer. The detachment forces were recorded after 30 s of contact between the polymer discs and a thin film of a 2% mucin solution. The results indicate that the mucoadhesive force values obtained for polymer blends HPMC:Carbopol fall within a relatively narrow range of 1.2–1.5 N. This behavior appears to reflect the intermediate adhesion profile resulting from the combination of the two polymers. Notably, a systematic trend was observed: increasing the proportion of Carbopol in the formulation HPMC:Carbopol led to a progressive increase in mucoadhesive strength. For matrices dominated by Carbopol, the mucoadhesive force was about 10% higher than that of the 1:1 (*w*/*w*) blend. The higher detachment forces observed for Carbopol-rich formulations may reflect composition-dependent differences in interfacial adhesion and matrix cohesion during contact with the model mucin layer. However, the measurements do not establish the specific molecular interactions or hydrated structures responsible for this behavior.

A similar trend was identified in HPMC:Kollidon systems. In this case, an increase in HPMC content was associated with a proportional rise in mucoadhesive strength. Specifically, doubling the HPMC content in the polymeric matrix resulted in a comparable twofold increase in detachment force. The increase in detachment force with HPMC content suggests a composition-dependent contribution to adhesion and/or cohesive resistance during contact with the model mucin layer. Hydrogen bonding and polymer chain interpenetration may represent plausible mechanisms [41]; however, neither these molecular interactions nor the formation of a continuous hydrated gel layer was directly demonstrated in the present study. Overall, the results demonstrate that polymer composition influences the measured detachment force and the ability of the matrices to maintain sufficient integrity during contact with the model mucin layer. However, the relative contributions of interfacial adhesion, hydration-related changes, and matrix cohesion cannot be quantitatively distinguished using the present experimental approach.

Preliminary control measurements performed using water without mucin, under the same experimental conditions, yielded substantially lower detachment forces than those obtained with the model mucin layer. The recorded values were generally at least one order of magnitude lower, with some approaching zero. These observations suggest that the presence of mucin contributed to the measured adhesion response. However, because water and the mucin solution differ in their physicochemical properties, the observed differences cannot be attributed exclusively to specific polymer–mucin interactions. Contributions from nonspecific adhesion, capillary forces, and matrix cohesion cannot be excluded.

### 3.5. Moisture Sorption Behavior of Mucoadhesive Polymeric Matrices

Moisture sorption measurements were conducted at 25 °C and 85% relative humidity (RH) to evaluate the hygroscopic behavior of the investigated polymeric matrices under controlled high-humidity conditions. Moisture uptake is an important consideration in the development and handling of polymer-based drug delivery systems [42]. The experimental conditions were selected to compare the susceptibility of different polymer compositions to moisture uptake rather than to reproduce physiological hydration conditions. Moisture sorption provides information on the ability of polymeric matrices to absorb water vapor from the surrounding environment. However, the resulting mass increase does not directly demonstrate dimensional swelling, gel-layer formation, or hydration behavior under liquid-immersion conditions. Therefore, the percentage moisture mass gain after 96 h was used as a comparative parameter (Table 3).

HPMC exhibited the lowest moisture mass gain among the investigated individual polymers. The incorporation of Carbopol or Kollidon into HPMC-based matrices increased moisture uptake, with the extent of mass gain depending on the polymer weight ratio. In both HPMC-based binary systems, increasing the HPMC fraction was associated with lower moisture sorption. These findings demonstrate that the hygroscopic behavior of the investigated matrices is strongly dependent on polymer composition under the applied experimental conditions. In contrast, Kollidon:Carbopol matrices exhibited similar moisture mass gain values across the investigated weight ratios, ranging from approximately 29.5% to 30.1% after 96 h (Table 3).

**Figure 7:**
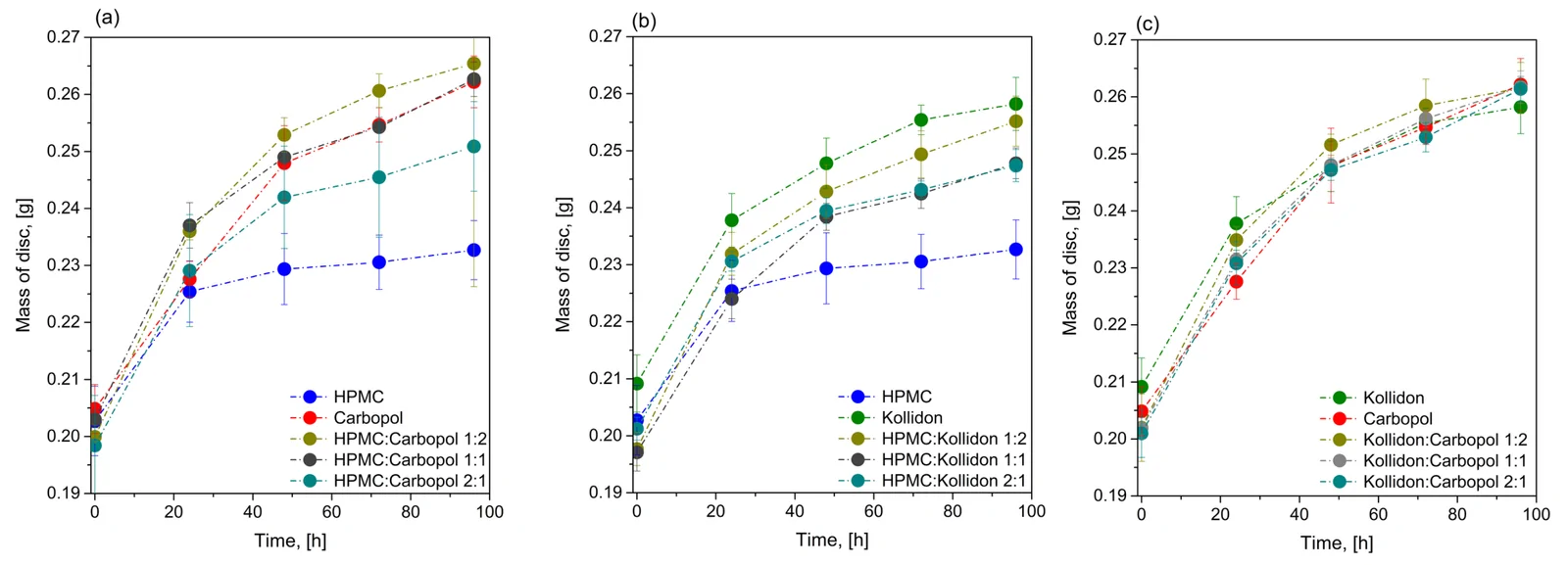
Moisture sorption profiles of individual polymers and their binary blends over a 4˗day period: (**a**) HPMC:Carbopol, (**b**) HPMC:Kollidon, (**c**) Kollidon:Carbopol. Measurements were conducted at RH = 85% and T = 25 °C using discs compressed at 2 MPa. Previously published data for individual polymers and HPMC-based 1:1 blends [30] were included for comparison with newly investigated formulations. Data are presented as means ± SD (*n* = 5).

Hydration and swelling are recognized factors affecting the mucoadhesive behavior of hydrophilic polymer systems [43], making moisture-related properties relevant to their physicochemical characterization. Figure 7 presents the moisture sorption curves for the polymer blends during storage in a climate chamber under controlled conditions.

For all analyzed mucoadhesive formulations, the most pronounced moisture uptake occurred within the first 24 h. The moisture sorption profiles of the Kollidon:Carbopol blends were similar across all tested compositions, with tablet mass gradually increasing to approximately 0.26 g and a near-linear mass increase continuing for over 48 h. In contrast, HPMC˗containing blends showed linear moisture uptake only during the first 24 h, followed by a marked slowdown at later time points. Previous studies involving HPMC-based polymer coatings have demonstrated that polymer composition influences water vapor sorption kinetics under humid conditions [44]. The subsequent reduction in the rate of moisture uptake observed for HPMC-based formulations in the present study may reflect changes in water sorption dynamics; however, the underlying mechanism cannot be established from these measurements alone. In contrast, Carbopol- and Kollidon-based systems exhibited a continued, albeit slower, increase in moisture uptake beyond 24 h. This extended absorption phase is likely associated with the highly hygroscopic nature of Kollidon, which retains water primarily through hydrogen bonding and surface adsorption [45], as well as the cross-linked structure of Carbopol, which undergoes progressive swelling and relaxation of its polymer network under sustained humidity exposure [46]. These mechanisms contribute to the prolonged sorption kinetics observed under high relative humidity conditions.

Moisture uptake by HPMC matrices occurred predominantly during the first 24 h, followed by a pronounced reduction in the rate of mass gain, suggesting an apparent plateau within the investigated time interval. In contrast, Kollidon and Carbopol matrices continued to absorb moisture throughout the experimental period, although the rate of sorption progressively decreased. After 96 h, the Kollidon matrix absorbed approximately 40–45 mg of moisture, whereas the Carbopol matrix exhibited a mass increase of approximately 50–55 mg. For the binary polymer blends, moisture sorption behavior depended on polymer composition. Kollidon matrices exhibited comparable moisture mass gain across the investigated weight ratios (approximately 29.5–30.1% after 96 h). Within the HPMC:Kollidon system, the Kollidon-rich formulation showed the highest moisture uptake, whereas in the HPMC:Carbopol system, the greatest moisture mass gain was observed for the Carbopol-rich blend. These findings demonstrate composition-dependent hygroscopic behavior under controlled humidity conditions without establishing dimensional swelling or the mechanical stability of hydrated matrices. Taken together, the results show that the functional behavior of mucin-interacting polymers is reflected in three complementary measured properties: initial wettability, moisture sorption, and detachment force against the model mucin layer. High wettability alone was not sufficient to ensure stable adhesion, as demonstrated by Kollidon-rich systems. In contrast, Carbopol-containing HPMC matrices provided a more favorable combination of wettability, moisture sorption, and measurable adhesion to the model mucin layer, highlighting the importance of polymer composition and weight ratio in designing functional biointerfacial matrices.

### 3.6. Limitations of the Study

Several limitations should be considered when interpreting the present findings. First, moisture sorption measurements conducted at 85% relative humidity provide information on hygroscopic behavior but do not directly characterize dimensional swelling or hydration under liquid-immersion conditions. Second, the OWRK-derived surface free energy values represent apparent, model-dependent estimates for heterogeneous and swellable polymeric surfaces. Formal uncertainty propagation from the measured contact angles to the OWRK-derived parameters was not performed. Therefore, small numerical differences between the calculated SFE values should be interpreted with caution and should not be considered conclusive evidence of differences in intrinsic surface energetics. Third, dry-state SEM observations do not establish the morphology or mechanical stability of hydrated matrices. Furthermore, disc thickness, apparent density, porosity, and mechanical strength were not independently quantified, limiting the ability to distinguish polymer composition effects from differences in compression-induced matrix structure.

Furthermore, although preliminary mucin-free control measurements indicated substantially lower detachment forces, differences in the physicochemical properties of water and the mucin solution, as well as variations in matrix cohesion, may also have contributed to the measured adhesion response. The experimental approach does not allow specific polymer–mucin interactions or individual adhesive and cohesive contributions to be conclusively identified. Complementary spectroscopic techniques (e.g., FTIR or Raman spectroscopy), rheological measurements, and appropriate interfacial analyses could further clarify the nature of polymer–polymer and polymer–mucin interactions and the properties of the hydrated matrices.

Finally, the present study was limited to short-term in vitro adhesion measurements using a model mucin layer. Long-term hydrated-state stability, drug release, and mucosal residence were not investigated. Additional studies are therefore required before the pharmaceutical performance of the selected formulations can be established.

## 4. Conclusions

This study demonstrated that the physicochemical and biointerfacial properties of compressed polymeric matrices are strongly governed by the polymer type and component weight ratio. The investigated HPMC-, Carbopol-, and Kollidon-based binary systems showed distinct wetting, moisture sorption, and mucin-layer adhesion profiles, confirming that the behavior of functional mucin-interacting polymer matrices cannot be predicted solely from the properties of the individual polymers. HPMC alone exhibited limited initial water wettability and relatively low moisture uptake, with an apparent plateau in moisture uptake during the investigated period. The incorporation of Carbopol or Kollidon generally improved the initial wettability of HPMC-based matrices and modified their moisture sorption behavior, depending on polymer composition. Carbopol-containing formulations exhibited measurable adhesion to the model mucin layer, although the molecular mechanisms responsible for this behavior were not directly established. In contrast, selected Kollidon-rich systems lost their structural integrity during adhesion testing, preventing reliable determination of the detachment force.

The moisture sorption measurements demonstrated composition-dependent differences in the hygroscopic behavior of the investigated polymer matrices. The Kollidon:Carbopol blends exhibited comparable percentage moisture mass gain values (approximately 29.5–30.1%) after 96 h, indicating that changes in their weight ratio had only a limited effect on moisture uptake under the applied conditions. Importantly, moisture sorption alone does not establish dimensional swelling or the cohesive stability of hydrated matrices. The loss of structural integrity observed for selected Kollidon-rich formulations during adhesion testing should therefore be considered a separate experimental observation rather than a direct consequence of the moisture sorption results. Therefore, an effective mucin-interacting polymer matrix should not only hydrate rapidly but also maintain sufficient cohesion to ensure stable contact with the mucin-containing environment.

Among the investigated formulations, the Carbopol-rich HPMC blend (1:2, *w*/*w*) emerged as a promising candidate for further investigation. Compared with the equimass HPMC:Carbopol (1:1, *w*/*w*) formulation, it exhibited a lower initial water contact angle (44.4° vs. 51.3°), higher apparent surface free energy (66.6 vs. 63.0 mJ/m^2^), and greater moisture mass gain after 96 h at 85% RH (approximately 32.7% vs. 29.4%). Furthermore, the Carbopol-rich formulation exhibited measurable adhesion to the model mucin layer under the applied experimental conditions. These combined results support its selection for further evaluation, although they do not demonstrate an optimal balance between hydration and matrix cohesion or establish long-term stability. The equimass HPMC:Carbopol (1:1, *w*/*w*) formulation also remains a relevant candidate for further investigation. In contrast, the loss of structural integrity observed for selected Kollidon-rich formulations during adhesion testing indicates a potential limitation of these compositions under the investigated conditions.

Overall, the results indicate that rational design of functional polymer matrices for mucin-containing environments requires simultaneous consideration of surface wettability, surface free energy, moisture sorption behavior, and mechanical stability under hydrated conditions. Polymer ratio tuning is therefore a useful strategy for optimizing the early interaction of polymeric matrices with mucin-containing media, but excessive hydration or Kollidon dominance should be avoided when stable and reproducible mucin-layer adhesion is required. These findings provide a physicochemical and biointerfacial basis for the selection of polymer ratios in future mucoadhesive formulation studies, while further API-loaded, ex vivo or in vivo validation is required before direct pharmaceutical performance can be claimed.

## Figures and Tables

**Table 1:** Surface tension (γ) of tested liquids: γ^p^—polar part of surface tension, γ^d^—dispersive part of surface tension; with a measurement uncertainty of ±0.2 mN/m.

Liquids	γ [mN/m]	γ^p^ [mN/m]	γ^d^ [mN/m]
Water (w)	72.8	51.0	21.8
Diiodomethane (d)	50.8	0	50.8

**Table 2:** Surface free energy (SFE) of polymers and their blends, estimated by the OWRK method; $\gamma_{\mathrm{sv}}^{p}$ is the polar component of SFE; $\gamma_{\mathrm{sv}}^{d}$ is the dispersive component of SFE; θ_w_ is the water contact angle, θ_d_ is the diiodomethane contact angle; polarity is the percentage part of polar component in surface free energy. Contact angles used for the OWRK calculations were measured 1 s after droplet deposition and are presented as mean values (*n* = 6; SD ≤ 3.5°).

Mucoadhesive Polymer		θ_w_ [deg]	θ_d_ [deg]	SFE [mJ/m^2^]	$\gamma_{\mathrm{sv}}^{p}$ [mJ/m^2^]	$\gamma_{\mathrm{sv}}^{d}$ [mJ/m^2^]	Polarity [%]
HPMC *		103.4	139.5	12.0	11.3	0.7	94.2
Carbopol *		47.2	20.8	64.1	16.4	47.6	25.6
Kollidon *		36.0	15.7	70.5	21.6	48.9	30.6
Blends polymers							
HPMC:Carbopol	1:2	44.4	13.4	66.6	17.2	49.4	26.0
	1:1 *	51.3	13.4	63.0	13.6	49.4	22.0
	2:1	55.9	15.6	60.4	11.4	49.0	19.0
HPMC:Kollidon	1:2	51.2	16.5	62.6	13.9	48.7	22.0
	1:1 *	53.9	19.6	60.7	12.8	47.9	21.0
	2:1	62.1	19.2	56.7	8.7	48.0	15.3
Kollidon:Carbopol	1:2	46.7	13.3	65.4	16.0	49.5	24.0
	1:1	42.7	14.4	67.3	18.1	49.2	27.0
	2:1	48.5	12.9	64.5	15.0	49.5	23.2

* Previously published experimental data from Ref. [30], included as reference values for comparison with the newly investigated formulations. All other formulation data were obtained in the present study.

**Table 3:** Percentage moisture mass gain of polymeric discs after 96 h of exposure to 25 °C and 85% relative humidity. Values are presented as means ± SD (*n* = 5).

Formulation	Weight Ratio (*w*/*w*)	Moisture Mass Gain After 96 h [%]
HPMC *	---	14.8 ± 0.5
Carbopol *	---	28.0 ± 0.4
Kollidon *	---	23.4 ± 0.5
HPMC:Carbopol	1:2	32.7 ± 0.9
HPMC:Carbopol *	1:1	29.4 ± 0.3
HPMC:Carbopol	2:1	26.5 ± 0.7
HPMC:Kollidon	1:2	29.1 ± 0.8
HPMC:Kollidon *	1:1	25.7 ± 0.3
HPMC:Kollidon	2:1	22.9 ± 0.6
Kollidon:Carbopol	1:2	29.5 ± 2.0
Kollidon:Carbopol	1:1	29.6 ± 0.8
Kollidon:Carbopol	2:1	30.1 ± 2.9

* Moisture sorption data previously reported in Ref. [30] are included for comparison with the newly investigated polymer compositions. Values represent the percentage mass gain after 96 h at 25 °C and 85% RH.

## Data Availability

All data can be obtained from the authors upon request.

## Supplementary Materials

The following supporting information can be downloaded at: https://www.mdpi.com/article/10.3390/polym18212681/s1, Figure S1: SEM images of compressed polymeric discs; Figure S2: Representative optical images of the upper surfaces of compressed polymeric discs; Figure S3: Wettability of polymer matrices measured after 1 and 15 s (darker and lighter color, respectively) of contact with media of different compositions; (a) HPMC:Carbopol (b) HPMC:Kollidon (c) Kollidon:Carbopol; Table S1: Representative chemical structures (repeating units) of HPMC, Carbopol 974P NF, and Kollidon VA 64 fine; Table S2: Chemical composition of experimental fluids; Table S3: Investigated polymer blends at various weight ratios; Table S4: One-way ANOVA results for 15 s contact angle values in different wetting media; Table S5: Two-way ANOVA results for 15 s contact angle values within each binary polymer family. SS–sum of squares; df–degrees of freedom; MS–mean square; F–F-statistic; pvalue–probability value. Significance codes: *** *p* < 0.001; ns—not significant; Table S6: Origin of experimental datasets included in the present study and their relationship to previously published results [30].
