# Supplementary material for: Composition-Dependent Wettability, Moisture Sorption and Adhesion of HPMC, Carbopol and Kollidon Binary Matrices to a Model Mucin Layer: An In Vitro Study
Source: Polymers (Basel). 2026 Oct 29;18(21):2681. doi: 10.3390/polym18212681 (PMC13766210; doi:10.3390/polym18212681)
Supplement: Supplementary file 1 [file polymers-18-02681-s001.zip › polymers-4635217-supplementary.pdf]

SUPPLEMENTARY MATERIAL

Composition-Dependent Wettability, Moisture Sorption and Adhesion of HPMC, Carbopol and Kollidon Binary Matrices to a Model Mucin Layer: An In Vitro Study

Table S1. Representative chemical structures (repeating units) of HPMC, Carbopol 974P NF, and Kollidon VA 64 fine.

Polymer	Chemical Structure
HPMC (hydroxypropylmethylcellulose)	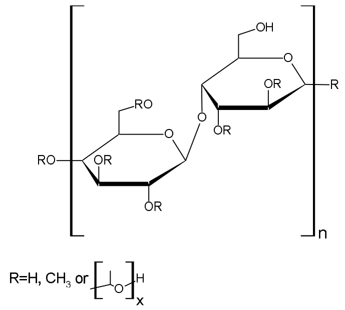 MW = 1· 10^4-1.5· 10^5
Carbopol 974P NF (corss-linked poly(acrylic acid))	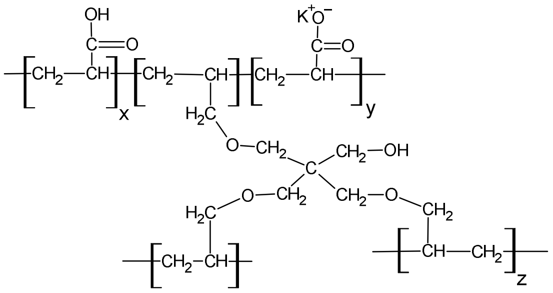 MW = 7·10^5 – 4·10^9
Kollidon VA 64 (vinylpyrrolidone-vinylacetate copolymer)	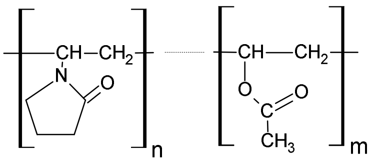 The ratio of n/m = 1.2;<br>n, m - number of monomer in polymer chain<br>MW = 4.5·10^4 – 7.0·10^4

Table S2. Chemical composition of experimental fluids.

**Medium**	**Components**	**Surface tension [mN/m]**	**pH**
SSF (Simulated Salivia Fluid without mucin)	• 8.0 g/L NaCl<br>• 0.19 g/L KCl<br>• 2.38 g/L KH_2PO_4<br>• 0.17 g/L $CaCl_2 \cdot 2H_2O$<br>• 0.2 g/L $MgCl_2 \cdot 6H_2O$<br>• Adjusted to pH 6.8 with NaOH	71.8	6.8
SSF_{muc} (Simulated Salivia Fluid with mucin; 0.1% mucin)	• SSF content +1 g/L mucin (w/v)	55.9	6.7
2% mucin	• 20 g/L mucin (w/v)	52.7	4.3*

*Literature/product-specification data for 2% aqueous porcine gastric mucin indicate pH 3.0–5.0; the exact pH of the medium used in this study was measured at 22 ± 1 °C before the experiments.

Table S3. Investigated polymer blends at various weight ratios.

Mucoadhesive blends	Composition weight ratio (w/w)
HPMC: Carbopol	1:2<br>1:1<br>2:1
HPMC: Kollidon	1:2<br>1:1<br>2:1
Kollidon:Carbopol	1:2<br>1:1<br>2:1

1. SEM images of selected binary polymeric discs

Supplementary SEM images of selected binary polymeric discs were included to illustrate the effect of component weight ratio on the surface morphology of compressed matrices. The HPMC: Carbopol and HPMC: Kollidon system were selected as a representative example because it showed clear composition-dependent differences in microstructure. The obtained micrographs indicate that variations in the relative proportion of both polymers are associated with changes in surface compactness, heterogeneity, and particle-domain organization. These features may affect the effective contact area with the wetting liquid and should therefore be considered as an additional morphological context for the interpretation of the wettability and hydration results presented in the main text.

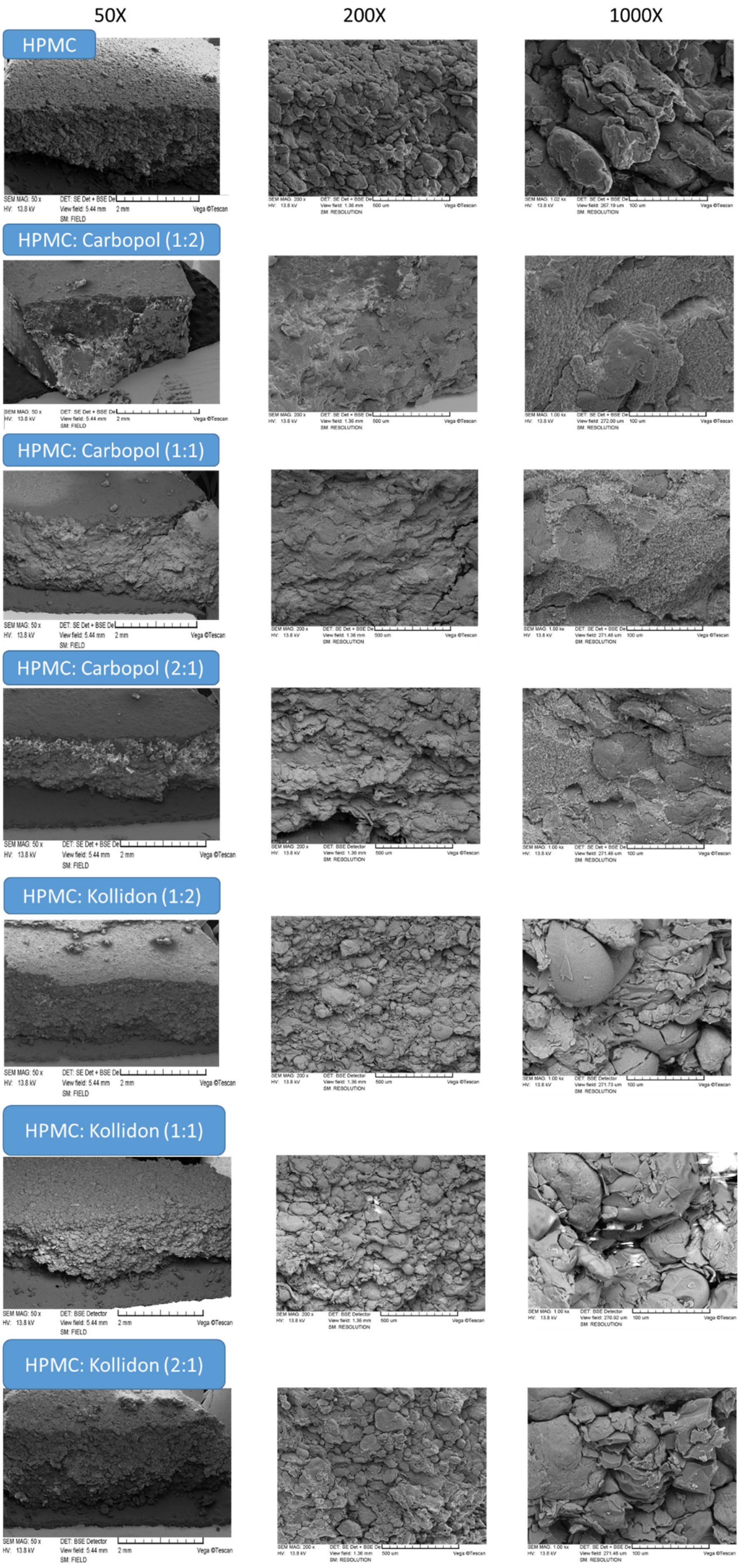
50X
200X
1000X
HPMC
HPMC: Carbopol (1:2)
HPMC: Carbopol (1:1)
HPMC: Carbopol (2:1)
HPMC: Kollidon (1:2)
HPMC: Kollidon (1:1)
HPMC: Kollidon (2:1)

Figure S1. SEM images of compressed polymeric discs

2. Surface of compressed polymeric discs

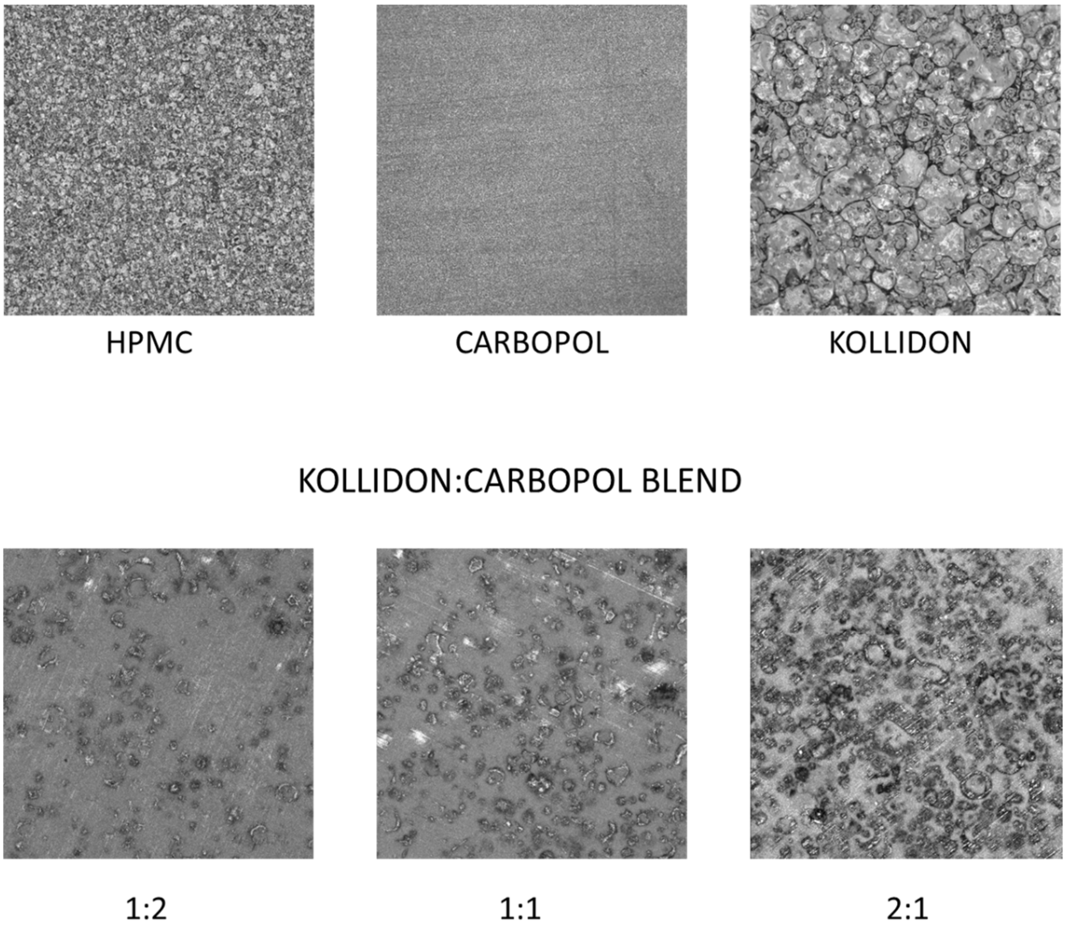

Figure S2. Representative optical images of the upper surfaces of compressed polymeric discs.

Images of Kollidon, Carbopol, and Carbopol:Kollidon matrices prepared at different weight ratios. The photographs illustrate visible differences in surface appearance and heterogeneity between formulations, which may contribute to differences in liquid spreading and contact angle measurements.

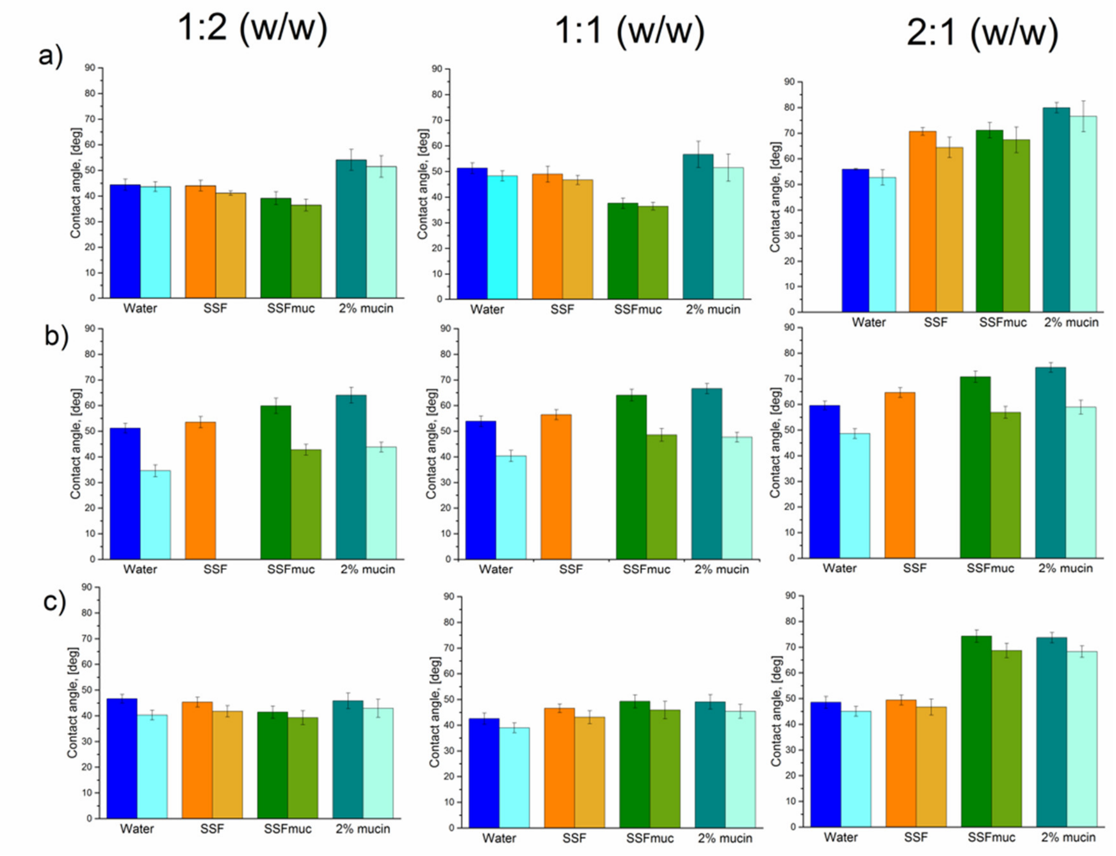

Figure S3. Wettability of polymer matrices measured after 1 and 15 s (darker and lighter color, respectively) of contact with media of different compositions; (a) HPMC:Carbopol (b) HPMC:Kollidon (c) Kollidon:Carbopol.

Table S4. One-way ANOVA results for 15 s contact angle values in different wetting media.

Medium	F-value	p-value	Interpretation
Water	303.92	<0.001	significant effect of formulation
SSF	996.41	<0.001	significant effect of formulation
SSFmuc	598.51	<0.001	significant effect of formulation
2% mucin	637.43	<0.001	significant effect of formulation

One-way ANOVA was performed separately for each wetting medium using the 15 s contact angle values. Differences were considered statistically significant at $p < 0.05$.

Table S5. Two-way ANOVA results for 15 s contact angle values within each binary polymer family. SS—sum of squares; df—degrees of freedom; MS—mean square; F—F-statistic; pvalue—probability value. Significance codes: *** $p < 0.001$; ns—not significant.

Polymer family	Effect	SS	df	MS	F	pvalue	Symbol	Significant_p<0.05
HPMC:Carbopol	Medium	2185,90	3	728,63	170,88	2,41E-29	***	yes
HPMC:Carbopol	Ratio (w/w)	6839,90	2	3419,95	802,06	5,12E-44	***	yes
HPMC:Carbopol	Medium × Ratio (w/w)	1170,84	6	195,14	45,76	1,38E-20	***	yes
HPMC:Kollidon	Medium	1019,28	3	339,76	188,35	1,75E-30	***	yes
HPMC:Kollidon	Ratio (w/w)	2524,06	2	1262,03	699,62	2,63E-42	***	yes
HPMC:Kollidon	Medium × Ratio (w/w)	23,19	6	3,87	2,14	6,14E-02	ns	no
Kollidon:Carbopol	Medium	1185,27	3	395,09	188,66	1,67E-30	***	yes
Kollidon:Carbopol	Ratio (w/w)	1920,75	2	960,38	458,58	4,42E-37	***	yes
Kollidon:Carbopol	Medium × Ratio (w/w)	1152,63	6	192,11	91,73	2,43E-28	***	yes

Interpretation

Two-way ANOVA was performed separately for each binary polymer family using the 15 s contact angle values, with wetting medium and polymer weight ratio as fixed factors. For HPMC:Carbopol matrices, both medium and polymer ratio significantly affected contact angle values, and a significant medium × ratio interaction was also observed. This indicates that the effect of the HPMC:Carbopol ratio on early wettability depended on the composition of the wetting liquid. A similar pattern was found for Kollidon:Carbopol matrices, where medium, ratio, and their interaction were all significant. In contrast, for HPMC:Kollidon matrices, both medium and ratio had significant effects, whereas the medium × ratio interaction was not

significant (p=0.061). This suggests that the ratio-dependent changes in wettability for HPMC:Kollidon were more consistent across the tested media.

Table S6. Origin of experimental datasets included in the present study and their relationship to previously published results [30].

Experimental analysis	Previously published data [30]	New experimental data (present study)
**Contact angle measurements and surface free energy (OWRK)**	Individual polymers (HPMC, Carbopol, Kollidon) and equimass HPMC:Carbopol and HPMC:Kollidon blends (1:1, w/w)	HPMC:Carbopol and HPMC:Kollidon blends at 1:2 and 2:1 ratios; Kollidon:Carbopol blends at 1:2, 1:1, and 2:1 ratios
**Adhesion to a model mucin layer**	Individual polymers and equimass HPMC-based blends (1:1, w/w), where reliable measurements were possible	Non-equimass HPMC-based blends (1:2 and 2:1, w/w) and Kollidon blends (1:2, 1:1, and 2:1, w/w)
**Moisture sorption (25 °C, 85% RH)**	Individual polymers and equimass HPMC:Carbopol and HPMC:Kollidon blends (1:1, w/w)	Non-equimass HPMC-based blends (1:2 and 2:1, w/w) and Kollidon:Carbopol blends (1:2, 1:1, and 2:1, w/w)
**Liquid uptake by polymer powders**	Not investigated	Liquid penetration measurements of individual HPMC, Carbopol, and Kollidon powders in different aqueous media
**Scanning electron microscopy (SEM)**	Not investigated	Morphological characterization of individual polymer powders and compressed binary matrices

Previously published experimental data [30] were included as reference datasets to enable comparison with the newly investigated polymer compositions. The present study extends the formulation range and introduces the Kollidon binary system. Reused data are not considered independent new measurements.
